# The Central Governor Model of Exercise Regulation Teaches Us Precious Little about the Nature of Mental Fatigue and Self-Control Failure

**DOI:** 10.3389/fpsyg.2016.00656

**Published:** 2016-05-04

**Authors:** Michael Inzlicht, Samuele M. Marcora

**Affiliations:** ^1^Department of Psychology, University of Toronto ScarboroughToronto, ON, Canada; ^2^Rotman School of Management, University of TorontoToronto, ON, Canada; ^3^Endurance Research Group, School of Sport and Exercise Sciences, University of Kent at MedwayChatham, England

**Keywords:** Self-control, motivation, exercise physiology, central governor model, ego depletion

## Abstract

Self-control is considered broadly important for many domains of life. One of its unfortunate features, however, is that it tends to wane over time, with little agreement about why this is the case. Recently, there has been a push to address this problem by looking to the literature in exercise physiology, specifically the work on the central governor model of physical fatigue. Trying to explain how and why mental performance wanes over time, the central governor model suggests that exertion is throttled by some central nervous system mechanism that receives information about energetic bodily needs and motivational drives to regulate exertion and, ultimately, to prevent homeostatic breakdown, chiefly energy depletion. While we admire the spirit of integration and the attempt to shed light on an important topic in psychology, our concern is that the central governor model is very controversial in exercise physiology, with increasing calls to abandon it altogether, making it a poor fit for psychology. Our concerns are threefold. First, while we agree that preservation of bodily homeostasis makes for an elegant ultimate account, the fact that such important homeostatic concerns can be regularly overturned with even slight incentives (e.g., a smile) renders the ultimate account impotent and points to other ultimate functions for fatigue. Second, despite the central governor being thought to take as input information about the metabolic needs of the body, there is no credible evidence that mental effort actually consumes inordinate amounts of energy that are not already circulating in the brain. Third, recent modifications of the model make the central governor appear like an all-knowing homunculus and unfalsifiable in principle, thus contributing very little to our understanding of why people tend to disengage from effortful tasks over time. We note that the latest models in exercise physiology have actually borrowed concepts and models from psychology to understand physical performance.

Self-control is important. Those with high self-control as kids tend to grow up to be happier, healthier, and richer adults who are less likely to commit crimes, abuse drugs, raise children as single-parents, or end up in prison (Moffitt et al., 2011). Self-control might be as important as being intelligent.

An unfortunate feature about self-control, however, is that is appears to wane over time (Baumeister et al., 2007), with people being less willing to exert effort the longer they have already exerted effort. Despite general agreement that people become fatigued, that effortful control has a refractory period, there is little agreement about *why* this is the case. Recently, a group of psychologists suggested that work from exercise physiology might offer clues.

Evans et al. (2015) integrated work from exercise physiology, specifically the literature on the central governor model of physical fatigue (St Clair Gibson and Noakes, 2004; Noakes, 2012), to offer a more complete picture of the nature of mental fatigue and self-control failure. While we admire the spirit of integration and the attempt to shed light on an important topic in psychology, our concern is that the central governor model is very controversial among exercise physiologists, with increasing calls to abandon it altogether (Weir et al., 2006; Marcora, 2008; Shephard, 2009). In this mini review, a collaboration between an exercise physiologist (Samuele M. Marcora) and a psychologist (Michael Inzlicht), we outline some problems with the central governor model, discussing how it might be unfit for psychology.

## Maintaining Effortful Control Over Time is Hard

In what is now a classic body of research, Baumeister et al. (1998, 2007) uncovered an important quality of self-control—it wanes over time. People do not tend to maintain self-control over long stretches of time, with self-control performance deteriorating with repeated exertions. This so-called ego depletion effect, whereby effortful control tends to fail when it follows previous bouts of effortful control, has been supported by over 200 separate studies (Hagger et al., 2010; but, see Carter et al., 2015; Inzlicht et al., 2015; Hagger et al., in press).

In addition to uncovering a basic detail about maintaining effort over time, Baumeister et al. (2007) offered an explanation based on the notion of limited resources. According to the limited resource model of control, effortful control relies on a limited resource that depletes quickly with use, such that initial exertions consume this resource, leaving less available for later use. So when people exert effortful control at Time 1, they consume this limited resource until the resource is exhausted such that people cannot control themselves at Time 2.

## Problems with a Resource Account of Self-Control

Despite the intuitive appeal of the resource account, many questions remain about how precisely depletion comes about (Inzlicht and Berkman, 2015). The first problem has to do with the precise identity of the resource underlying control. Although initial research on glucose indicated that it might be *the* resource underlying effortful control (Gailliot et al., 2007), these findings have proven very controversial, being challenged on multiple grounds (Kurzban, 2010; Beedie and Lane, 2012; Molden et al., 2012).

A second problem is the increasing number of studies that are inconsistent with a strict resource account (Inzlicht and Schmeichel, 2012; Masicampo et al., 2014). If self-control wanes over time because of the literal depletion of some resource that has run out, or that threatens to run out, it is difficult to understand how simple incentives can turn this effect around (Muraven and Slessareva, 2003). If the resource that underlines self-control is exhausted by a previous task, how is it possible that thinking about one of your core values (Schmeichel and Vohs, 2009), receiving a surprising gift (Tice et al., 2007), or focusing on one’s ongoing feeling states (Saunders et al., 2016) can instantly restore the resource and, thus, self-control?

Both problems suggest that self-control’s refractory period is not satisfactorily explained by accounts that rely on the notion of limited resources and that it might be better explained by models where motivation plays a central role. A number of related such models have emerged, and while each emphasizes different things, they all converge on the notion that self-control is the product of priorities, motives, and values that change dynamically over time (Kurzban et al., 2013; Inzlicht et al., 2014; Kool and Botvinick, 2014).

Though these motivational accounts do a better job of explaining the existing data, the ultimate explanation for *why* self-control wanes over time is somewhat unsatisfying. While we suspect that ultimate accounts based on the notion of maintaining balance between various goals and avoiding opportunity costs (Kurzban, 2016; Francis and Inzlicht, in press) might offer biologically plausible ultimate accounts, phenomenologically, it feels like we run out of energy when we are fatigued, even if such conceptions are historically new (Hockey, 2013). Certainly, the argument goes, dwindling energy supplies must play some part in the feeling of fatigue. And this is where the central governor model comes in.

## The Central Governor Model

The central governor model has the attractive quality of taking into account information about both energy supply and motivational state (Noakes, 2012). As such, Evans et al. (2015) offer it as a way out of the current impasse. Given the clear role that motivation plays (Inzlicht et al., 2014) and the intuitive appeal of notions about dwindling energy, the idea of a central governor seems like a perfect fit. In brief, the central governor is proposed to be some central nervous system mechanism that takes as input information about energetic needs, current physiological states, and various motivational drives to regulate physical exertion to save the organism from catastrophic homeostatic failures during physical exertion (Noakes et al., 2005). The argument here is that fatigue stemming from physical and mental exertion may not be separate systems, with the latter co-opting the pre-existing neural machinery of the former. Critically, the model offers a plausible ultimate explanation for why self-control seems to wane over time. Without a central governor that throttles physical effort, people might exert themselves to the point of hurting themselves and causing serious bodily damage. No bodily harm is caused by mental exertion, but the same throttle mechanism is thought to be at work.

Despite being attractive, we worry that the idea of a central governor is only appealing on its surface. Just as with the resource model it is trying to supplant, the central governor model is biologically implausible and, worse, unfalsifiable, appearing more and more like some sort of all-knowing homunculus. We make this claim for three reasons.

First, while the preservation of bodily homeostasis during exertion makes for an elegant ultimate account, the fact that such important homeostatic concerns can be overturned with even small changes in motivational value renders the ultimate account toothless. If even mild incentives, such as subliminally presented smiling faces (Blanchfield et al., 2014b) can reverse people’s tendency to slacken physical effort, to what extent can effort—especially mental effort—meaningfully threaten homeostasis and cause bodily harm to begin with? Second, despite the central governor being thought to take as input information about the metabolic needs of the body, there is no credible evidence that mental effort (i.e., thinking) actually consumes inordinate amounts of energy (Raichle and Mintun, 2006). To what extent, then, do energy considerations play a role in throttling mental effort? Third, the most recent version of the central governor (Noakes, 2012), is now so all-encompassing that it cannot be falsified by any possible array of data.

## Problems with the Governor

The principal claim of the central governor model is that the brain dynamically and subconsciously regulates physical exertion by afferent inputs from the body, thereby allowing for the completion of a given task in a safe manner (Noakes et al., 2005). Crucially, this subconscious regulation of physical exertion is enacted to serve the ultimate function of preventing homeostatic breakdown and serious bodily damage. For example, during intense exercise the central governor is thought to prevent myocardial ischemia (Noakes, 1997), which results from insufficient blood flow to the heart that can lead to damage in the heart muscle. Evans et al. (2015) suggest mental exertion might be implemented by the same neural circuitry as physical exertion, with mental exertion co-opting or exapting the machinery of the central governor to serve the same ultimate function: prevent homeostatic breakdowns. In the case of mental effort, the central governor is ostensibly there to prevent profligate energy use and to prevent strains to various biological systems, including the cardiovascular and immune systems (Muraven et al., 2006; Segerstrom and Nes, 2007).

### If Biological Concerns Are So Crucial, How Can They Be So Readily Abandoned?

Despite the intuitive appeal of a system working for the ultimate function of preventing homeostatic breakdown, it is difficult to see evidence of this ultimate function at work given just how easily this ultimate function is overturned by motivational incentives, and how little actual energy is required for mental effort.

If the central governor is truly there to prevent homeostatic breakdown, surely simple incentives like motivational self-talk should have no impact (Blanchfield et al., 2014a). Indeed, an earlier version of the central governor model explicitly stated that conscious override was not possible or desirable: “the presence of conscious over-ride would be undesirable because it would increase or maintain the exercise intensity, thereby threatening homoeostasis” (Noakes et al., 2005, p. 121). If homeostasis is the ultimate function, then motivational override makes little sense. However, it is now abundantly clear that motives matter a great deal, with people being able to push themselves to evermore heights of physical endurance. Indeed, even when people physically exert themselves to the point of exhaustion, they are able to exert themselves even more if sufficiently motivated (Marcora and Staiano, 2010; McCormick et al., 2015). The same holds for mental effort (Boksem et al., 2006).

While it is true that motivation has now been incorporated as an important input into the workings of the central governor (Noakes, 2012), and it is this modified central governor that forms the basis of Evans et al.’s (2015) model, such motivational override is thought to only occur during life-threatening situations or other extreme circumstances (e.g., high-stakes sports competition). The idea here is that the consequences of motivational override are so extreme—including myocardial ischemia, torn muscles, ruptured tendons, broken teeth, even death—that override should occur very rarely and only in extreme circumstances. In reality, motivation, even slight and subtle motivation (e.g., Blanchfield et al., 2014a), appears perfectly capable of regularly overriding this supposed ultimate function.

If something as slight as the viewing of a subliminal smiling face can get people to exercise for longer (Blanchfield et al., 2014b), in what way can we say that the governor is driven by some all-important ultimate function that should only be overridden in extreme circumstances? It is clear that people can override the after-effects of mental fatigue by subtle inducements, like interacting with someone who is polite (Muraven et al., 2008); it is also clear that perceptions of effort are more consequential than actual effort in determining mental performance over time (Clarkson et al., 2010; Werle et al., 2014). Such findings are inconsistent with the notion that the application of mental effort or feelings of depletion are governed by physical considerations (i.e., homeostatic signals from the body).

In contrast, the body contains a number of veritable subconscious control systems to preserve homeostasis that cannot be consciously overridden. For example, the sudomotor control system, which controls sweating, is a subconscious system that functions to preserve thermal homeostasis. No matter how motivated one is not to sweat—for example, at a job interview or on a first date—the sudomotor system will make you sweat when you feel hot. Motivation cannot override this veritable homeostatic system, just like motivation should not be able to override any subconscious homeostatic system controlling the exertion of effort. In our view, the fact that even subtle motives can override the supposed hard limits of the central governor suggests that the determinants of fatigue and subjective effort cannot be based on sensing depleted energy sources or other sensory signals from the body.

### Does the Central Governor Modulate Exertion Based on Available Energy?

An attractive feature of the central governor model is that it is thought to incorporate information about bodily needs for energy (as well as motivation) in modulating effortful exertion. Effort is throttled, in other words, to conserve energy for future use. This only makes sense, however, if mental energy actually consumes meaningful amount of energy. There is no credible evidence supporting this, however, (Kurzban, 2010). Despite muscular claims that mental effort leads to meaningful drops in glucose (Gailliot et al., 2007), it is now clear that these claims are biologically implausible. Studies using Positron Emission Tomography (PET), which offer direct measurement of glucose metabolism in the brain, suggest that while the brain consumes lots of energy, most of this energy consumption is for intrinsic, basal metabolism that is just as evident during rest as during mental effort. More critically, relative to this high rate of basal metabolism, the amount consumed by evoked tasks is remarkably low (Raichle and Mintun, 2006), with some suggesting that effortful tasks might increase glucose consumption by at most 0.2 calories (Kurzban, 2010), an amount readily circulating and available in the brain. Thus, even supporters of the resource model (e.g., Baumeister, 2014) now concede that there is very little evidence supporting the notion that mentally effortful tasks consume any more energy than non-effortful tasks (e.g., Marcora et al., 2009).

If mental effort does not meaningfully deplete energy, of what use is a central governor that supposedly monitors energy expenditure? In our view, such energy monitoring is superfluous and makes the central governor redundant with models that talk mostly about motives and goals (e.g., Marcora, 2008; Inzlicht et al., 2014; Kool and Botvinick, 2014). While Evans et al. (2015) discuss many other bodily signals that feed into the central governor to determine the amount of effort to exert—including signals about cortisol levels, immune system activity, thermal conditions of the body, and so on—recent research makes clear that perception of effort is not directly determined by such afferent bodily signals (Marcora, 2009), but instead might reflect activity in premotor and/or motor areas of the brain (Marcora, 2008; de Morree and Marcora, 2015).

### Can the Central Governor Model be Falsified?

One of the things that separates scientific theories from non-scientific theories are that the former can be falsified (Popper, 2005). Although there are problems with falsificationism, especially when we ask if a specific theory has been falsified by any one specific empirical observation (Godfrey-Smith, 2003), a theory needs to be falsifiable in principle. That is, in order to determine the quality of a theory we need to place it at risk of being disconfirmed; and only when a theory withstands multiple such risks, can we say that the theory is a good one.

In our view, the central governor model is not falsifiable. It did not start out that way, however. In the original formulation of the theory, the central governor was said to be a subconscious system that could not be overridden by conscious forces, lest it threaten homeostasis (Noakes et al., 2005). This is a falsifiable statement because if one finds that conscious motives can override states of fatigue, one can falsify the theory. And, it is now abundantly clear that conscious override exists, thereby falsifying the theory (McCormick et al., 2015). Instead of abandoning the theory, however, proponents of the central governor simply amended the model such that conscious override is now possible, with motivation being one of many determinants of physical exertion. As we note above, the problem with this sleight of hand is that it now undermines the very function of the central governor, to guard against homeostatic breakdown during exertion.

Worse it makes the theory unfalsifiable in principle: “The prediction of this model is that potentially **everything**…can potentially affect athletic performance” (Noakes, 2012, p. 6, emphasis in the original). If a model suggests that everything can determine how long someone mentally and physically perseveres, can such a model ever be demonstrated to be false? Can such a model ever offer specific insights to understanding why physical or mental effort is aversive and why people tend to avoid it? In our view, the central governor resembles the fabled homunculus and is another theoretical soup stone (Navon, 1984)—it appears to do theoretical work, but upon closer inspection it actually does very little.

## Conclusion

Making a connection between mental fatigue and physical fatigue is laudable and we agree that much can be learned from such cross-fertilization. The problem, in our view, is that the central governor model is controversial at best and unfalsifiable at worst. It offers an ultimate account of fatigue that is disconfirmed in the most mundane of situations and, given present knowledge of physiology, it is not clear if it can deliver what it promises.

Bridging the gap between mental and physical fatigue is a laudable goal. There is already well established and increasingly accepted work in exercise physiology that has looked to psychology to understand the factors that determine disengagement during physical tasks (Marcora, 2008; Marcora and Staiano, 2010; McCormick et al., 2015). This psychobiological model based on motivational intensity theory (Brehm and Self, 1989; Gendolla and Richter, 2010) suggests that perception of effort and potential motivation are the central determinants of task engagement, with people consciously deciding how much or how little effort to apply based on a number of considerations. We believe that the gap between mental and physical fatigue is not very broad, but we would rather not bridge it with a flawed model that is increasingly out of favor.

## Author Contributions

MI and SMM wrote and edited the mini-review.

## Conflict of Interest Statement

The authors declare that the research was conducted in the absence of any commercial or financial relationships that could be construed as a potential conflict of interest.
